# Earliness *Per Se* by Temperature Interaction on Wheat Development

**DOI:** 10.1038/s41598-019-39201-6

**Published:** 2019-02-22

**Authors:** Helga Ochagavía, Paula Prieto, Meluleki Zikhali, Simon Griffiths, Gustavo A. Slafer

**Affiliations:** 1 Department of Crop and Forest Sciences and AGROTECNIO (Center for Research in Agrotechnology), University of Lleida, Av. Rovira Roure 191, 25198 Lleida, Spain; 2 John Innes Centre, Norwich Research Park, Colney Ln, Norwich, NR4 7UH United Kingdom; 3 ICREA, Catalonian Institution for Research and Advanced Studies, Barcelona, Spain; 4 Present Address: Seed Co Limited, Rattray Arnold Research Station, PO Box CH142, Harare, Zimbabwe

## Abstract

Differences in time to heading that remain after photoperiod and vernalisation requirements have been saturated are classified as earliness *per se* (*Eps*) effects. It has been commonly assumed that *Eps* genes are purely constitutive and independent of environment, although the likely effect of temperature on *Eps* effects in hexaploid wheat has never been tested. We grew four near isogenic lines (NILs) for the *Eps* gene located in chromosome 1D (*Eps-D1*) at 6, 9, 12, 15, 18, 21 and 24 °C. In line with expectations we found that lines carrying the *Eps*-late allele were always later than those with *Eps*-early alleles. But in addition, we reported for the first time that the magnitude of the effect increased with decreasing temperature: an *Eps* x temperature interaction in hexaploid wheat. Variation in heading time due to *Eps* x temperature was associated with an increase in sensitivity to temperature mainly during late reproductive phase. Moreover, we showed that *Eps* alleles exhibited differences in cardinal (base, optimum, maximum) temperatures and that the expression of *ELF3*, (the likely candidate for *Eps-D1*) also interacted with temperature.

## Introduction

Differences in time to flowering are critical for wheat adaptation and the improvement of yield potential^1–4^. Genotypic variation in the developmental rate during phases preceding anthesis is mainly caused by differences in sensitivity to photoperiod and vernalisation^5,6^, the genetic pathways of which are interconnected^7^, and allow coarse-tuning of adaptation^8^.

However, there are still relatively minor variations in flowering time once requirements of vernalisation and photoperiod are totally satisfied. These differences are regulated by earliness *per se* (*Eps*) genes^9,10^. Their effects are usually small^8,11^, and critical for the fine-tuning of developmental patterns^10,12,13^. There is a large degree of variation in *Eps* genes in germplasm adapted to different regions^14,15^. The genetics of *Eps* is not as well understood as it is for *Ppd* and *Vrn* effects^10^, and the underlying genes and causal polymorphisms have only been identified very recently in hexaploid wheat. In *Triticum monococcum* L., a cereal ortholog of *Arabidopsis thaliana* circadian clock regulator *LUX ARRHYTHMO/PHYTOCLOCK* 1 *(LUX/PCL*1*)* was proposed as a promising candidate gen for the earliness *per se* 3 (*Eps-3A*^*m*^) locus^16^ and the ortholog circadian clock regulator *EARLY FLOWERING 3* (*ELF3*) was identified as a candidate gene for the earliness *per se Eps-A*^*m*^1 locus^17^. *ELF3* was suggested to be the best candidate gene within the *Eps-D1* locus in *Triticum aestivum* as a deletion containing *ELF3* is associated with advanced flowering and altered expression of an evening loop member *GIGANTEA* (*GI*)^18^. In this work, we used the same near isogenic lines (NILs) developed in that study^18^ and studied as well the expression of both *ELF3* and *GI*, as explained in Materials and Methods (see below).

It has been assumed that earliness *per se* is “constitutive” and, therefore, independent of the environment^10,14^ (that is why it was termed ‘*per se*’). However, it was hypothesised that these *Eps* effects would likely be temperature sensitivity genes^9^. Temperature has a universal effect on wheat development in that all phases and all cultivars develop faster under high than under low temperatures^19^. This universal effect can be seen well beyond wheat in developmental rates of other plant species as well as of other living organisms not controlling their own temperature^20,21^. Therefore, all phases are sensitive to temperature but this does not necessarily imply that there is no variation in sensitivity^22^. Working with an *Eps* gene in *Triticcum monococcum* (that had an unusually large effect on time to heading; *c*. 60 d difference between lines with the *Eps-A*^*m*^*1*-late and -early alleles)^12^, the *Eps* x temperature interaction was confirmed^12,23,24^. Furthermore, in genetic terms it has been shown that *ELF3* may have an important role in temperature entrainment^25,26^ which suggested that changes in clock gene expression in a 24 hours period were associated with daily temperature changes^26,27^.

More recently, we grew several *Eps* NILs derived from Spark x Rialto hexaploid wheats under field conditions^28^, that had also been tested in the UK^11,29^. It was seen that the magnitude of the *Eps* effects were stronger in the experiments conducted in the UK than in Spain. This was interpreted as a possible effect of temperature on the action of *Eps* alleles in hexaploid wheat, as it had been shown for *Eps-A*^*m*^*1* gene in *T. monococcum* (see above).

Although there is evidence that ambient temperature affects different developmental traits^22,30^, the specific interaction between *Eps* genes and temperature beyond time to heading in hexaploid wheat has not been reported. A more comprehensive understanding on whether particular sub-phases of “time to heading” are sensitive to *Eps* and *Eps* x temperature is essential, because it is during these pheno-phases when sources and sinks contributing to yield are being determined^2^.

In this study we aimed to quantify *Eps* x temperature interactions, for the first time (i) in hexaploid wheat considering *Eps* alleles (i.e. shown to possess significant effects on time to heading)^11,28^, (ii) considering independently the effects on different developmental processes, using NILs derived from the same Spark x Rialto cross from which we identified performance differences in Spain and UK, and (iii) estimating the cardinal temperatures (maximum, optimum and minimum) for lines carrying the *Eps-D1* early and late alleles. In addition, we also studied in two of the temperature regimes, the expression of the circadian clock gene *ELF3*, which had been identified as the candidate gene for *Eps-D1* in these NILs^18^ and *GI*, which is thought to be the major target of *ELF3* in the flowering time pathway.

## Results

### Abnormalities in development

**Figure 1:**
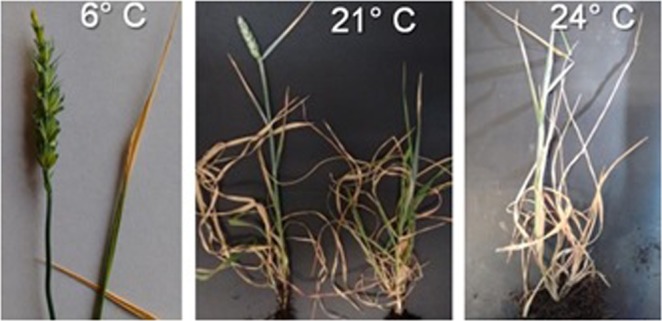
Abnormalities in development in the plants grown at 6, 21 and 24 °C. At the lowest temperatures plants headed but anthers were not extruded, at 21 °C more than half of the plants of NILs with early alleles headed (left image) while none of the plants reached heading in the NILs with the late allele (right image) and at 24 °C none of the plants progressed in their development to heading. Pictures were taken at the end of the experiment.

When plants were grown at the lowest and two highest temperature regimes (6, 21 or 24 °C) the patterns of development exhibited abnormalities, none of which were visible plants grown under the other thermal conditions (9, 12, 15, and 18 °C) (Fig. 1).

Plants grown at 6 °C never reached anthesis in spite of developing quite normally, though expectedly slowly, until heading. This remained the case 89 d after heading of the NILs carrying the *Eps*-late alleles. Lack of anthesis was not an effect of temperature on the genes controlling anther extrusion. Florets inside the spikelets grown under this thermal regime developed abnormally: e.g. the anthers lost their colour and the florets seemed to have been dehydrated before they could become receptive for pollination (dehydration is a major criterion used to establish that a floret has died)^31^. In addition, apical spikelets started to lose their normal green colour after heading. Consequently, although 100% of the plants reached heading normally, none reached anthesis when grown at the lowest temperature of the study (Supplementary Fig. S1).

When grown at 21 °C none of the plants with *Eps*-late alleles reached heading, whilst *c*. 60% of those with *Eps-*early alleles did, though only less than half of them continued developing normally to reach anthesis (Fig. S1). However, all plants of both genotypes (with *Eps-*early and –late alleles) reached the terminal spikelet stage normally; indicating that the impairment of development occurred during the late reproductive phase. At 24 °C all plants, regardless of the *Eps* allele they carried developed normally up until the appearance of the first 5–6 leaves. Leaves produced after that appeared extremely slowly and did not reach full size. These events were concurrent with premature senescence of the earlier leaves. In terms of floral development, none of the plants reached the double ridge stage after 55 days (equivalent to a 1320 °C d assuming a Tb = 0 °C) since the onset of the experiments and at this point, the plants were dying.

In the rest of the results section, we considered only the plants that exhibited normal development for the particular trait considered. Due to the relatively large failure in reaching anthesis from plants that had reached heading normally, we used heading (rather than anthesis) as the most integrative developmental trait. However, for all conditions in which plants reached anthesis normally, heading time was very well correlated with time to anthesis with a slope higher than 1 (1.38 ± 0.10) due to the fact that the durations are in days and all phases, including that from heading to anthesis, are delayed at lower temperatures (Fig. S2). Although the very high coefficient of determination is due to the clear effect of temperature, differences between NILs also contributed.

### Phenology

**Figure 2:**
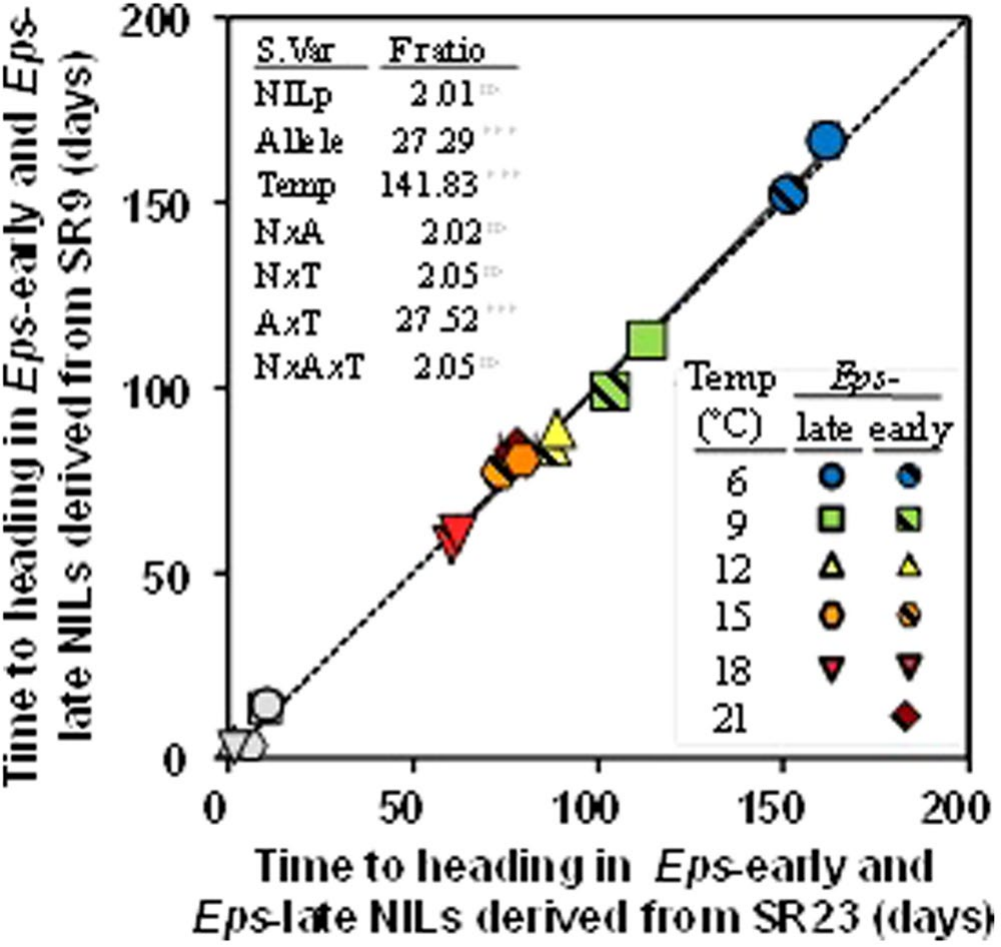
Time from the initiation of the experiment to heading for the NILs carrying the same *Eps* alleles in each of the two NIL pairs (NILs derived from either SR9 or SR23, see Materials and Methods) under the temperature regimes in which heading was reached. Dashed line is the 1:1 relationship. Solid line fitted by linear regression (R^2^ = 0.993, *P* < 0.001). Grey smaller data-points (bottom left corner) stand for the differences in time to heading between lines with the *Eps*-late or -early alleles (i.e. the delay produced by the *Eps*-late respect to its *Eps*-early counterpart); which are not considered in the regression. Inset are the F-ratio values of the main factors and their interactions (*** and ns indicates that the F-ratio was significant, *P* < 0.001, and not significant, respectively). Segments on each symbol stand for the SEs (if not seen is because the magnitude was smaller than the size of the symbol).

The plants having the same *Eps* alleles from the two pairs of NILs, derived from either SR9 or SR23, displayed exactly the same behaviour. Considering time to heading as the overarching trait embracing developmental processes in this study, it is easy to see that the *Eps-*early NILs of each of the two families (SR9 and SR23) reached heading almost simultaneously at each of the temperature conditions, and the same was true for the *Eps-*late NILs of both pairs (Fig. 2). This shows that genetic background effects of the NIL pair (whether lines were derived from SR9 or SR23), as well as its interaction with temperature, were not significant (Fig. 2, inset). In fact, the linear regression of the relationship in Fig. 2 was not only very highly significant but also had an intercept (−2.23 ± 3.01 d) and a slope (1.03 ± 0.03) which were not significantly different from 0 d and 1, respectively. Therefore, in the rest of the paper the results of the two *Eps-*early and the two *Eps-*late NILs are shown as an average.

**Figure 3:**
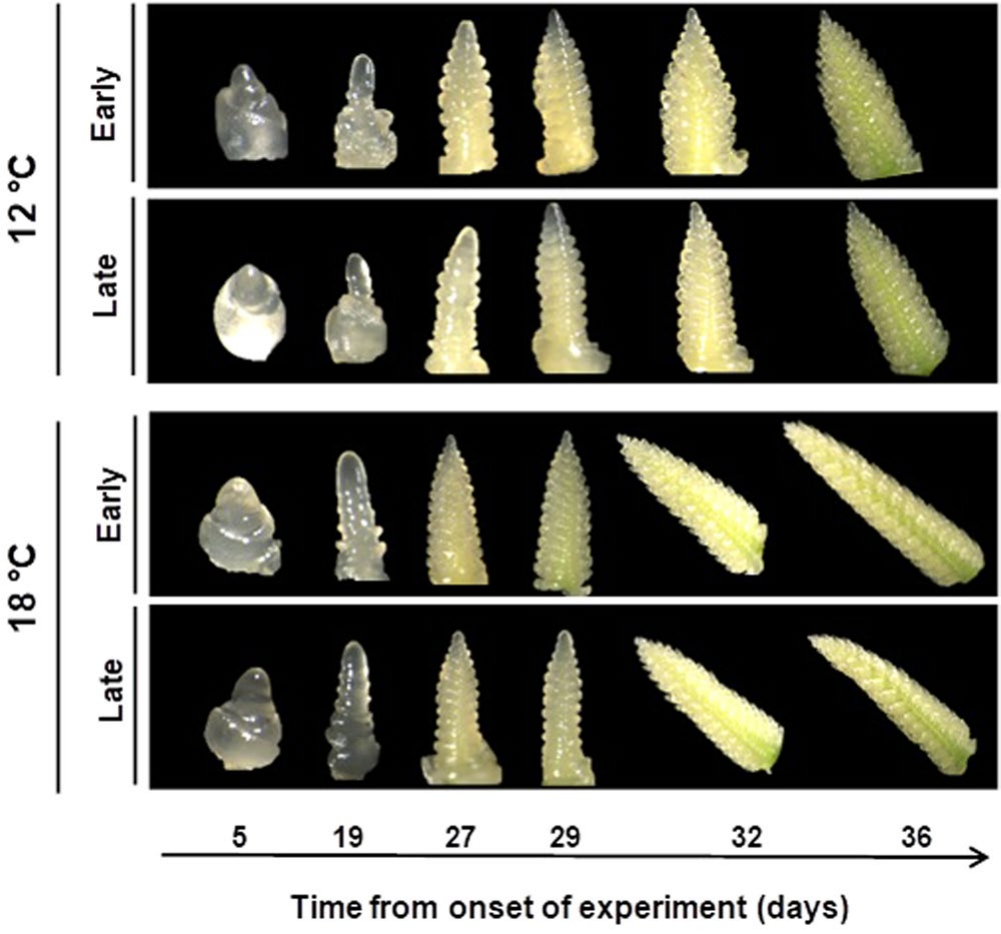
Illustration of apex development in selected lines representative of each sample from 5 to 36 d after the onset of the experiment for lines carrying *Eps-*early and -late alleles grown at constant temperature of 12 (top panel) and 18 °C (bottom panel).

In general, it was clear that both higher temperatures and the *Eps*-early alleles accelerated development, as reflected by their F-ratios (with the effect of temperature much larger than that of the *Eps* alleles and *Eps* x temperature interaction; Fig. 2, inset). In order to illustrate these results visually, the dissected apexes observed by microscope at regular intervals in NILs carrying the *Eps-*early or -late alleles at 12 and 18 °C were shown in the Fig. 3. We found that apexes developed at 18 °C were more advanced than those grown at 12 °C within each of the same lines. Moreover, within the same temperature regime, lines carrying the *Eps-*early allele were more developed than those with the *Eps-*late allele (Fig. 3).

**Figure 4:**
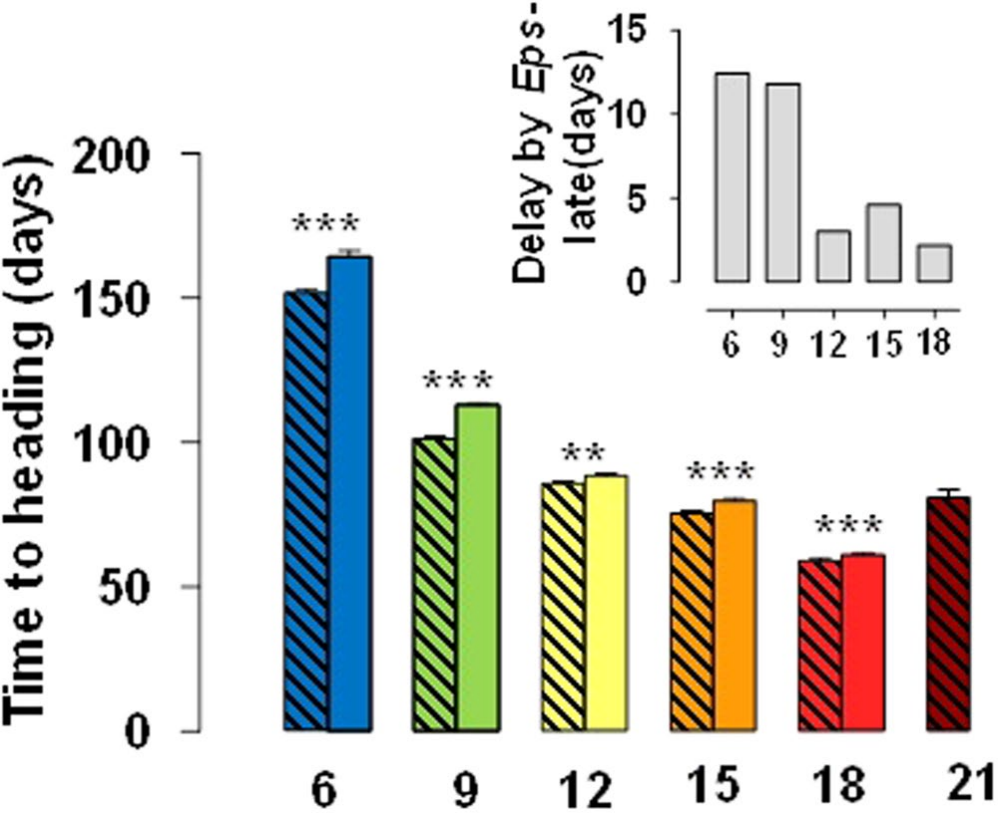
Days from the onset of the experiment to heading for lines carrying *Eps-*early (hatched bars) or *Eps-*late (solid bars) alleles grown under constant temperatures of 6, 9, 12, 15, 18, and 21 °C (treatment at 24 °C was not included as none of the plants at that condition reached heading). Inset is a detail of the difference between lines with the *Eps-*late or *Eps*-early alleles (for the range of temperatures, 6 to 18 °C, in which plants of both types of NILs reached heading). Asterisks indicate the statistical significance of the differences between lines with late and early alleles at each temperature from the LSmeans contrast (**P < 0.01, ***P < 0.001).

Between 6 °C and 18 °C, increased temperature accelerated the developmental processes and consequently reduced the period from onset of the experiment to heading (Fig. 4). However, when comparing the time to heading under 21 and 18 °C, the opposite was true: plants of NILs with the *Eps-*early allele headed earlier at 18 than at 21 °C (Fig. 4) and, as already stated, those carrying the late allele did not reach heading at all.

It was also clear that lines carrying the *Eps-*late allele were consistently later than those with the *Eps-*early allele (Fig. 4). However, the magnitude of the difference was not the same across temperatures: at low temperatures the effect of the *Eps* gene was more pronounced than at mild and warm temperatures (Fig. 4, inset); and this interaction was still evident if the delay produced by *Eps-*late allele is considered in relative terms. At 21 °C the difference was even qualitative as early lines reached heading while late lines did not.

This revealed a clear *Eps* x temperature interaction of quantitative nature: i.e. there was a difference in the magnitude of the effect, but the interaction was not crossover in nature as lines with the *Eps-*late allele were never earlier than those carrying the *Eps-*early allele (Fig. 4). In addition, the no crossover interaction was reflected by the fact that the F-ratio of the *Eps* x temperature interaction was much smaller than that of temperature (Fig. 2, inset).

**Figure 5:**
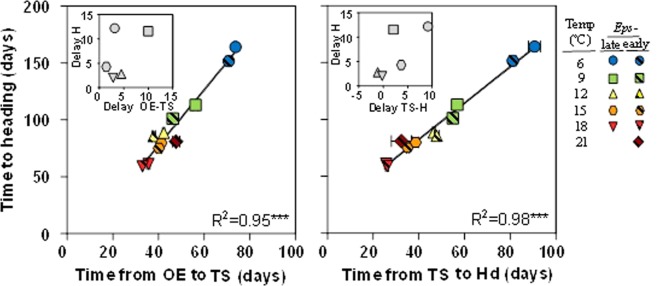
Relationship between time to heading and either the period from the onset of the experiment (OE) to terminal spikelet (TS) (left panel) or the late reproductive phase from TS to heading (Hd) (right panel) for lines carrying *Eps*-early (hatched symbols) or *Eps-*late (solid symbols) alleles grown under constant temperatures of 6, 9, 12, 15, 18 and 21 °C. Lines fitted by linear regression (*P < *0.001*). Inset each panel is a detail of the delay produced by the *Eps-*late allele (difference between lines with the *Eps-*late or *Eps*-early alleles) in time to heading respect to the delay in the component phases considered in the study.

Across all treatments, time to heading was well explained by the two component phases considered in the study: from the onset of the experiment to terminal spikelet (Fig. 5, left panel), and from then to heading (Fig. 5, right panel). Naturally, the very high coefficients of determination are due to the universal effect of temperature: as all phases are sensitive. However, the sensitivity of both phases was not identical: the time elapsed between the onset of the experiment and terminal spikelet ranged from 33 to *c*. 74 d (an increase of 220% in duration of the leaf and spikelet initiation phase from 18 to 6 °C), whilst duration of the period from terminal spikelet to heading ranged from *c*. 26 to *c*. 91 d (an increase of 350% in duration of the late reproductive phase from 18 to 6 °C).

In addition, the delay produced by the *Eps-*late allele (difference between plants carrying *Eps-*late and *Eps-*early alleles) in time to heading was better explained by the delay in the late reproductive phase (Fig. 5, inset right panel) than by that of the period from the onset of the experiment to terminal spikelet (Fig. 5, inset left panel).

Thus, the effect of the main factors (temperature and *Eps* alleles) as well as that of the *Eps* x temperature interaction shown in time to heading was more clearly reflected in the effect these treatments had on the rate of development during the late reproductive phase than during the period from the onset of the experiment to terminal spikelet.

### Developmental responses to temperature

The significant *Eps* gene x temperature interaction in duration of phases implies that the temperature sensitivity of developmental rates during different phases must vary between NILs carrying *Eps-*early and -late alleles.

**Figure 6:**
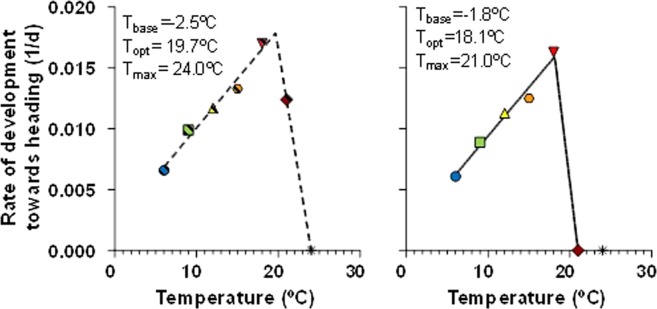
Relationship between the rate of development between the onset of the experiment and heading and temperature for lines carrying *Eps*-early (left panel) or *Eps*-late (right panel) alleles grown under constant temperatures of 6, 9, 12, 15, 18, 21 and 24 °C. To avoid a biased estimate of parameters, lines were fitted by bi-linear regression fitted with data at temperatures between the base and the maximum thresholds (for lines carrying *Eps*-early 6–24 °C and for *Eps*-late 6–21 °C). Insets are the calculated cardinal temperatures (base, T_base_; optimum, T_opt_; and maximum, T_max_) for each group of NILs.

We found that the rate of development towards heading was very well fitted to a bi-linear regression (R^2^ = 0.993 and 0.807 for *Eps-*early and -late alleles, respectively). The relationship had a first positive slope (indicating that plants develop faster at higher temperatures, within the range from the base and the optimum thresholds) followed by a second negative one (indicating decline in developmental rates with increasing temperatures between the optimum and maximum thresholds) (Fig. 6). The parameters of the relationship revealed slight changes in base, optimum and maximum temperatures, with the NILs carrying *Eps-*late alleles showing slightly lower optimum temperature, lower maximum temperature and slightly higher base temperature (Fig. 6). Moreover, the initial slopes (i.e. sensitivity to temperature between base and optimum values) were clearly different, indicating that the rate of development towards heading was more sensitive in the *Eps-*early (0.807 10^−3^ [d °C]^−1^, whose reciprocal is 1239 °C d) than in the *Eps-*late NILs (0.720 10^−3^ [d °C]^−1^, whose reciprocal is 1389 °C d). This differential sensitivity to temperature seemed to be the mechanistic bases for the delay produced by the *Eps-*late alleles in reaching heading.

Regarding the rates of development during the two phases considered in this study as components of time to heading we found that the general differences between *Eps-*late and *Eps-*early lines for time to heading were maintained (Fig. S3). Interestingly, the base temperature was noticeably increased from the early phase until terminal spikelet to the later phase from then to heading. The base temperature estimated for the whole period from the onset of the experiment to heading (Fig. 6) reflected well a weighted average of the base temperatures estimated for two phases composing time to heading (Fig. S3).

The difference between NILs in slope was negligible for the phase from the onset of the experiment to terminal spikelet (*c*. 1.19 10^−3^ [d °C]^−1^, whose reciprocal is *c*. 840 °C d for both *Eps*-late and -early NILs; Fig. S3A,B). On the other hand, the rate of development during the late reproductive phase was much more sensitive to temperature in the *Eps*-early (2.08 10^−3^ [d °C]^−1^, whose reciprocal is *c*. 481 °C d; Fig. S3C) than in the *Eps*-late NILs (1.69 10^−3^ [d °C]^−1^, whose reciprocal is *c*. 592 °C d; Fig. S3D).

### Gene expression over a 24 h period

Although in this study, we analysed the level of expression of *ELF3* from the three (A, B and D) genomes, we still found differences of expression between *Eps-D1* early lines with *ELF3*-loss function and late lines with alleles from Rialto. Hence, the expression of *ELF3* related with the A and B genomes did not compensate the expression linked to the D genome.

**Figure 7:**
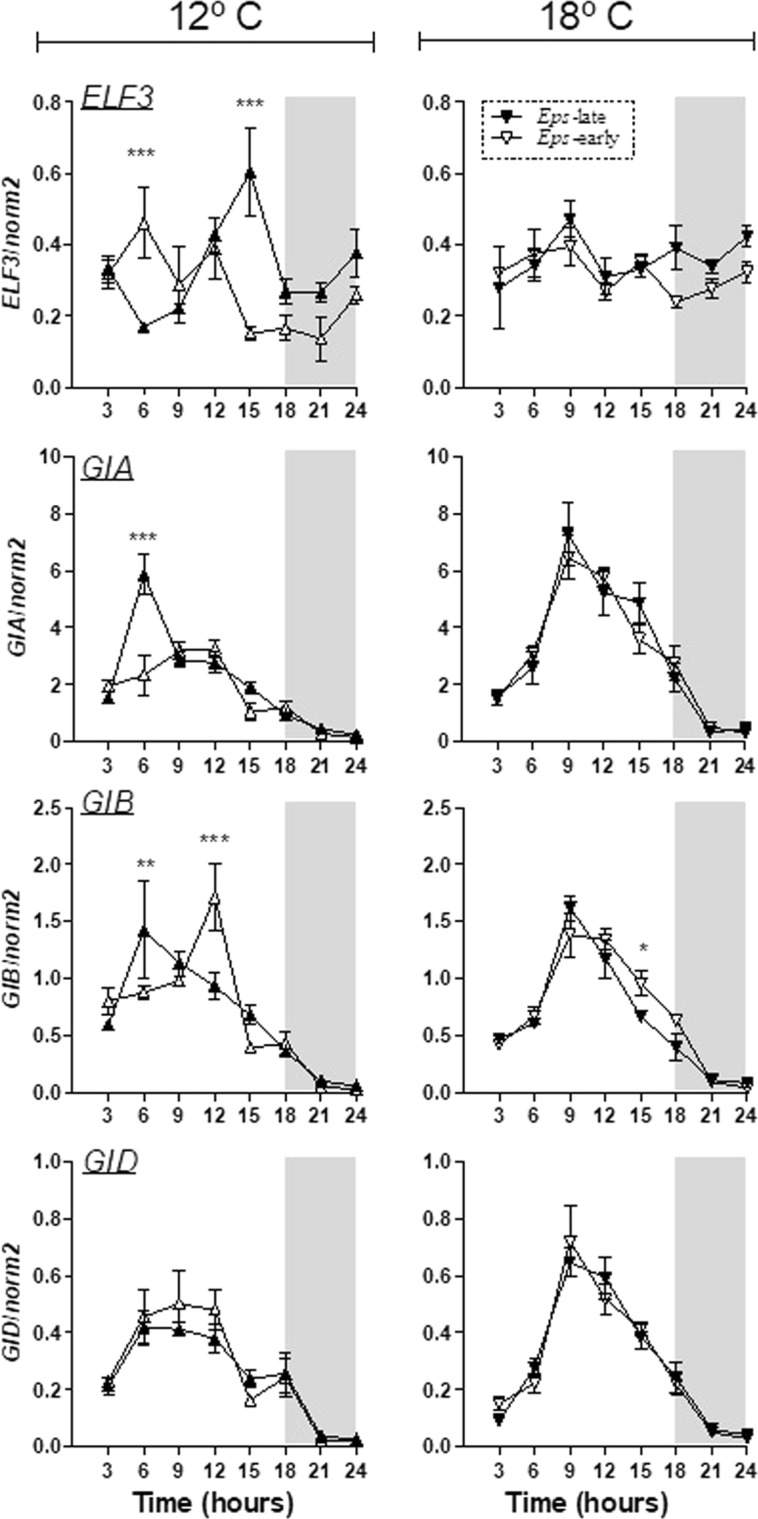
Expression patterns of genes *ELF3* (generic primer amplifying all three A, B and D homoeologues), *GIA*, *GIB* and *GID* homoeologues *Eps-* early (open symbols) and *Eps*-late (closed symbols) genotypes. The vertical axis represents the relative expression of the genes against the house keeping gene *norm2*. Dark period is indicated by grey square. Asterisks indicate the statistical significance of the differences between lines with *late* and *early* alleles at each temperature from the LSmeans contrast (*P < *0.05*, **P < *0.01*, ***P < *0.001* and no asterisks means that the differences was not significant).

Virtually no significant differences in the expression of *ELF3* or *GI* were found between late and early flowering NILs at 18 °C (Fig. 7). However, at 12 °C the lines are clearly differentiated by the circadian expression profiles of these genes (Fig. 7). *ELF3* expression at 12 °C actually shows an increase in total *ELF3* transcript at six hours after dawn in early lines, this is reversed by hour 15 at which time *ELF3* expression reaches its lowest level of expression in early flowering lines, a state which was maintained into the dark period and is consistent with an early flowering phenotype as *ELF3* is a floral repressor. This pattern is mirrored in the *GI* expression profiles at 12 °C; this time with 6 hours significantly increased expression levels of *GIA* and *GIB* in late lines and followed by an increase in *GIB* for early flowing lines at 12 hours.

## Discussion

The fact that temperature, between the base and the optimum thresholds, accelerated development was expected, as there is a huge body of literature reporting this effect in wheat^19,32^ as well as not only in other crops^33^ but also in other completely unrelated organisms^20,21^. The overall delay in time to heading produced by the introgression of *Eps-*late alleles was also expected, as these lines were selected because of their previous reported effects in field conditions both in the UK^11^ and in Spain^28^; conditions in which the actual effect of *Eps* alleles can be challenged, as they can be masked by the presence of other major developmental genes^11,34^. But the work described here contributes more specifically by providing the first time direct evidence of the *Eps* x temperature interaction in hexaploid wheat. Previous reports indicated that cultivars may differ in their sensitivity to temperature^22,30^ as well as *Eps* x temperature interactions but working with an *Eps* gene of *T. monococcum* usually with strong effects^12,23,24^; but to the best of our knowledge this interaction have never been shown before for an *Eps* gene with an expected subtle effect on time to heading in bread wheat.

**Figure 8:**
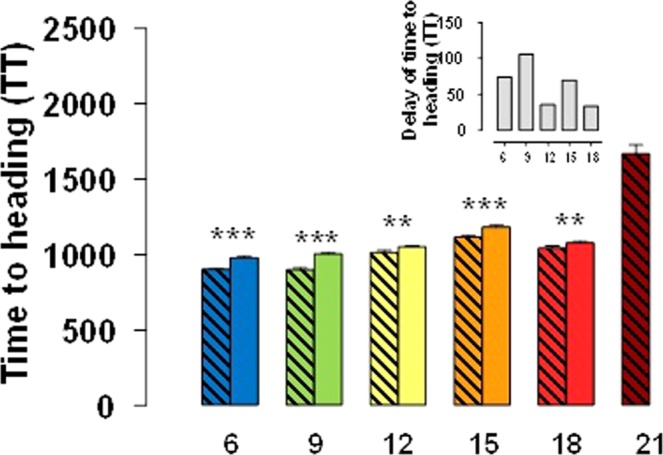
Thermal time from the onset of the experiment to heading (estimated using 0 °C as base temperature) for lines carrying *Eps-* early (hatched bars) or *Eps-*late (solid bars) alleles grown under constant temperatures of 6, 9, 12, 15, 18, and 21 °C. Inset is a detail of the difference (in °C d) between lines with the *Eps-*late or *Eps*-early alleles. Asterisks indicate the statistical significance of the differences between lines with late and early alleles at each temperature from the LSmeans contrast (**P < *0.01*, ***P < *0.001*).

Indeed the magnitude of the deceleration of development produced by introgressing *Eps-*late alleles was stronger at relatively low temperatures below the optimum threshold. The fact that the difference in time to reach heading between NILs carrying the *Eps-*late and -early alleles was affected by temperature was not due to a simple effect of temperature itself on the “extra time” required by the *Eps*-late NILs. Actually, if we analyse the results in thermal time using a general base temperature of 0 °C (Fig. 8), the same interaction is still noticeable. Naturally the effect of temperature is lost for the range 6–18 °C, and the thermal time increases at 21 °C because this temperature was higher than the optimum.

This is in agreement with the apparent inconsistency of the *Eps* genes effects on wheat development under field conditions of Spain^28^ and UK^11^. When facing the fact that the effects of *Eps-D1* were stronger in relatively cool British conditions than in rather warmer Spanish fields, we hypothesised that the reason was the difference between locations in ambient temperature^28^. In the present study, we provided evidences that support that hypothesis.

It was demonstrated then that the *Eps-D1* gene studied in this work was likely to control sensitivity to temperature; and the *Eps-*early and –late alleles determine different degrees of sensitivity to this environmental cue, rather than being an intrinsic factor modifying the rate of developmental processes independent of the environment. Furthermore, the fact that the sort of interaction we reported did not involve crossover offers some confidence that this allele could be an important tool for wheat breeding programs in which pursue to fine-tuning time to heading (at least it would not be expected to result in changes in rank order of expected results, as suggested may be the case with other *Eps* genes, which might exhibit stronger interactions)^9^.

It seemed clear that the reduction in time to heading produced by the introgression of the *Eps-*early allele from chromosome 1DL of Spark into that of Rialto was mainly associated with an increase in sensitivity to temperature during the late reproductive phase. We are not aware of other studies reporting these effects for *Eps* genes in hexaploid wheat, but the finding is consistent with the fact that different phases of wheat and barley development may respond to temperature with varying degrees of sensitivity^22,30^. This capacity of *Eps-D1* to modify time to heading through modifying development during stem elongation is important, as in most cases it is assumed that the effects of *Eps* genes are limited to (or at least are stronger in) the earlier phases of crop development^9,12^. Deployment of alleles that affect the developmental rates of the late reproductive phase may be instrumental as they can be used to not only fine-tuning heading time but also the likely fertility of the spikes. Optimizing the duration of this developmental phase may be an avenue for further improving the likelihood of floret primordia to become fertile florets^35^ that would further improve the number of grains^36,37^, which is the yield component most largely affecting yield^2,38,39^.

The earliness *per se* locus (*Eps-D1*) studied in this research was suggested to have as candidate gene, the circadian clock gene, *ELF3*^18^. For this reason, we analysed the expression of this gene under two different temperatures in order to explore if expression of this circadian clock gene was modulated by temperature. In fact, we ascertained that the deletion on 1D that contained *ELF3* was associated always to “Spark” allele and linked with early flowering genotypes in both temperatures. These results are in agreement with previous evidences that *ELF3* repressed the gibberellin (GA) biosynthesis^40^, which are able to promote the transition from vegetative to reproductive development. In addition, we also confirm *ELF3* repressed the expression of the evening loop gene *GI*^18,41,42^ and that these differences in expression were clear at 12 °C but largely absent at 18 °C. It was shown that *ELF3* expression was decreased throughout the diurnal cycle in early flowering NILs under a temperature regime of 16–18 °C in the light and 13–15 °C in the dark period^18^. The current study might indicate that the cooler dark temperatures might have been crucial for the induction of differences in heading date and gene expression in that study and that gene expression studies in more dynamic field conditions might shed more light on the observed *Eps* x temperature interaction. Previous studies have reported that *ELF3* plays a role on temperature entrainment of the clock in *Arabidopsis*^25^ and in barley^26^. In temperature entrainment, the daily rhythms of the circadian clock are synchronized with daily changes of the temperature. In our studies, daily temperature changes were inexistent because we applied constant temperatures (12 or 18 °C) but we found different responses of *ELF3* to temperature, level of expression increased as temperature increased. This might indicate that *ELF3*, besides having an effect on the temperature entrainment, as it was described previously, also has a role in temperature compensation^43^. Another study showed that the binding of *ELF3* to target promoters was dependent on temperature^44^ providing a plausible mechanism for the temperature interaction effects seen here.

## Materials and Methods

### Growth conditions

The experiments were carried out in the facilities of both the University of Lleida (UdL, Spain) and John Innes Centre (JIC, UK) using growth chambers (GER-1400 ESP, Radiber SA, Spain and controlled environment room, respectively). One single seed was sown in pots (200 or 400 cm^3^, at the UdL and JIC, respectively) filled with a mixture of 30% peat and 70% soil (UdL) or cereal mix compost composed of 40% peat and 40% soil and 20% grit (JIC). Before starting the experiments in the growth chambers, all seedlings were vernalised at 4 °C for 49 days. To maximise uniformity at the onset of the experiment (i.e. when pots were transferred from the vernalisation room to the growth chambers), we sowed 50–100% (depending on availability of seeds) extra plants of each line. Then, at the end of the vernalisation period, we selected those that showed the same stage to avoid noise caused by variability arising from uneven development among plants at the commencement of the study. After vernalisation, the plants were transferred to the corresponding growth chambers. At this time, the seedlings were at stage 1.27 ± 0.21 leaves of the scale of Haun^45^.

Chambers were always set under long day conditions (18 hours). Pots inside the chambers were rearranged approximately once a week to minimise the effects of likely differences in microenvironment at different positions within each chamber.

### Treatments

The treatments consisted of a factorial combination of two sets of near isogenic lines (NILs) carrying different *Eps* alleles and seven temperature regimes.

- NILs were developed at John Innes Centre (Norwich, UK) from two double haploid lines derived from the cross Spark × Rialto (SR9 and SR23, both carrying the *Eps*-early allele of Spark in chromosome 1DL) as described in Zikhali *et al*.^11^. In brief, each of these doubled haploid lines was back-crossed twice with the recurrent parent Rialto (which carries the *Eps*-late allele in 1DL) producing, within each of the two families, BC_2_ progenies (equivalent to BC_3_ in terms of recurrent parent background composition). These BC_2_ lines were self-pollinated producing BC_2_F_3_ families, and within these families lines homozygous for *Eps* alleles on chromosome 1DL (carrying the *Eps*-early or -late) were selected using SSR markers. Each NIL pair was then composed of a NIL carrying *Eps*-late allele and another carrying *Eps*-early allele, within the families derived from the SR9 and SR23 lines.
- Temperature treatments were kept constant in for each chamber and covered a range from 6 to 24 °C at intervals of 3 °C. The temperature regimes of 24, 21, 15, 9 and 6 °C were imposed at the UdL whilst those of 18 and 12 °C were at JIC.

Fifty-five (UdL) or 63 (JIC) pots per genotype were arranged inside each chamber (temperature regime) in a completely randomised design with three replicates. Each replicate was formed by 1/3 of the pots corresponding to each NIL, that is, 18 (UdL) and 21(JIC) pots per replicate and per genotype, resulting a total of 220 (UdL) and 252 (JIC) pots at each temperature regime.

### Gene expression analyses over a 24 hours period

Expression analysis of *ELF3* (with generic primer amplifying all three A, B and D homoeologues), *GIA*, *B* and *D* homoeologues were measured in both NILs pairs (*Eps*-early and -late alleles) on plants grown at 12 and 18 °C. The authors studied the expression of *GI* as well as *ELF3* because, in *Arabidopsis* at least, the influence of *ELF3* on flowering time is achieved by direct repression of *GI*^46,47^. Samples were collected every 3 hours over a 24-hours period. Three whole plants per genotype and treatment were immediately frozen in liquid nitrogen and crushed. Crushed plant material were store at −80 °C until the RNA extraction step. RNA extraction was carried out by using RNEasy Plant Mini Kit (Quiagen) with TRI reagent according with John Innes Centre Standard Operating Procedure. The concentration of RNA extracted was measured through spectrophotometry by NanoDrop 1000 (Thermo Scientific). From the concentration of RNA (ng/µl) obtained by NanoDrop 1000, the volume (µl) needed to get 5 ng of RNA was calculated for each sample. H_2_O was added to make up to 8 µl of total volume. The DNA was removed from the RNA sample by adding 1 µl of buffer DNase I recombinant RNase-free, 1 µl of DNase I recombinant RNase-free, 1 µl of dNTPs at a concentration of 10 Mm (Promega UK LDT) and 1 µl of random primer mix were then added and a standard PCR was run by thermal cycler MJ Research PTC-225 Peltier with forty cycles that included denaturation for 20 s at 95 °C, annealing for 20 s at 55 °C and polymerization at 72 °C for 1 min. After the forty cycles, the PCR was held at 72 °C for 5 mins. Of the resulting cDNA we took 2.5 µl to which it was added 10 µl of GoTaq qPCR Master Mix (Promega UK LDT), 1 µl of forward primer and 1 µl of reverse primer whose sequences were AGCGATTTCCAGCTGCCTTC and TGCGAAGAGGCCAGTCAGTC (the primer is termed as *norm2* and it was used previously)^18^. Analysis of relative transcription levels of the respective sequences was performed by using LightCycler software.

### Measurements

Three plants per replicate of each genotype (all in all 9 plants per genotype) were labelled at the beginning of the experiments. On all plants we measured (i) the phenological stages of seedling emergence (stage DC10), heading (DC59) and anthesis (DC65) following the Decimal Code^48^. Additionally, timing of terminal spikelet^49^ was determined from periodical dissection under binocular microscope sampling at each time one plant per treatment and replicate from the remaining plants (15 and 18 plants used for periodic dissections at UdL and JIC, respectively).

### Statistical analysis

Data of variables were subjected to analysis of variance and the relationships between traits were analysed through regression analyses. All analyses were performed using the statistical software JMP^®^ Pro Version 12.0 (SAS Institute Inc. Cary, NC, USA). Differences among *Eps* alleles in each temperature treatment were tested using ANOVA and a *post-hoc* analysis of LSMeans Contrast.

## Supplementary information

SUPPLEMENTARY FIGURES

## Data Availability

The datasets generated during and/or analysed during the current study are available from the corresponding author on reasonable request.
