# Supplementary material for: Earliness Per Se by Temperature Interaction on Wheat Development
Source: Sci Rep. 2019 Feb 22;9:2584. doi: 10.1038/s41598-019-39201-6 (PMC6385287; doi:10.1038/s41598-019-39201-6)
Supplement: Supplementary file 1 — SUPPLEMENTARY FIGURES [file 41598_2019_39201_MOESM1_ESM.pdf]

EARLINESS *PER SE* by TEMPERATURE INTERACTION ON WHEAT DEVELOPMENT

Helga Ochagavía[1], Paula Prieto[1], Meluleki Zikhali[2,†], Simon Griffiths[2], Gustavo A. Slafer[1,3,*]

[1] Department of Crop and Forest Sciences and AGROTECNIO (Center for Research in Agrotechnology), University of Lleida, Av. Rovira Roure 191, 25198 Lleida, Spain
[2] John Innes Centre, Norwich Research Park, Colney Ln, Norwich NR4 7UH, United Kingdom
[3] ICREA, Catalonian Institution for Research and Advanced Studies, Spain

[†]Present Address: Seed Co Limited, Rattray Arnold Research Station, PO Box CH142, Harare, Zimbabwe

*Corresponding author: slafer@pvcf.udl.cat

SUPPLEMENTARY FIGURES

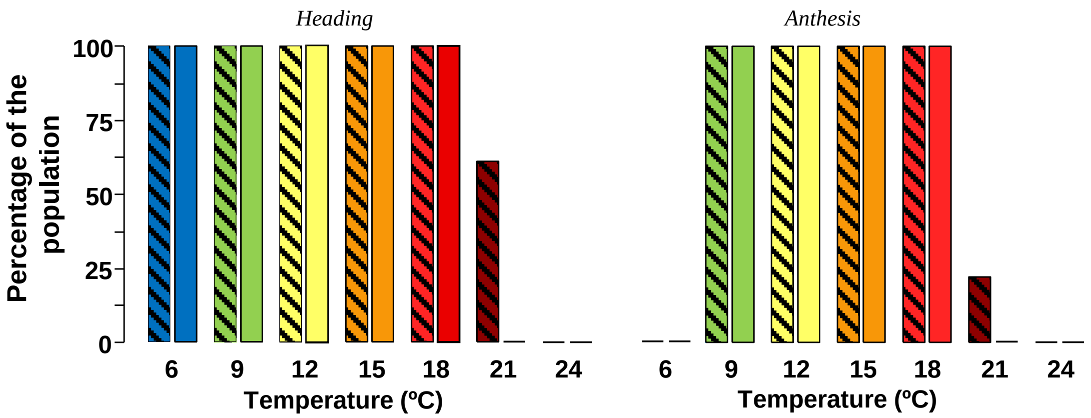

Supplementary Figure S1. Percentage of the population of plants with *Eps*-early (hatched bars) or *Eps*-late (solid bars) that reached heading (left) or anthesis (right) grown under constant

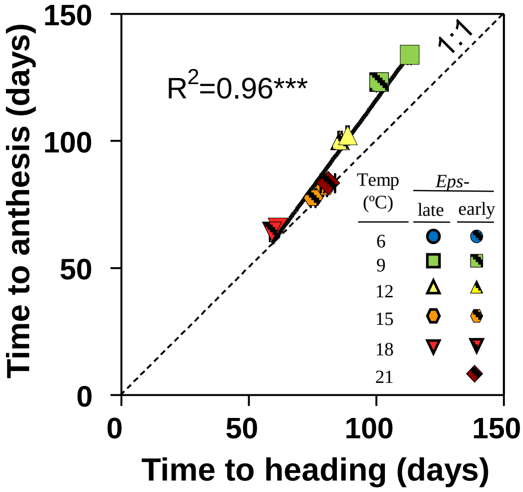

Supplementary Figure S2. Relationship between time to anthesis and time to heading for lines carrying *Eps*-early (solid symbols) or *Eps*-late (hatched symbols) alleles grown under constant temperatures of 9, 12, 15, 18, and 21ºC (in the latter temperature only lines with *Eps*-early alleles). Treatments at 6 and 24°C (all lines) and lines with *Eps*-late alleles at 21ºC were not included as none of the plants at those conditions reached anthesis. Segments on each symbol stand for the SEs (if not seen is because the magnitude was smaller than the size of the symbol).

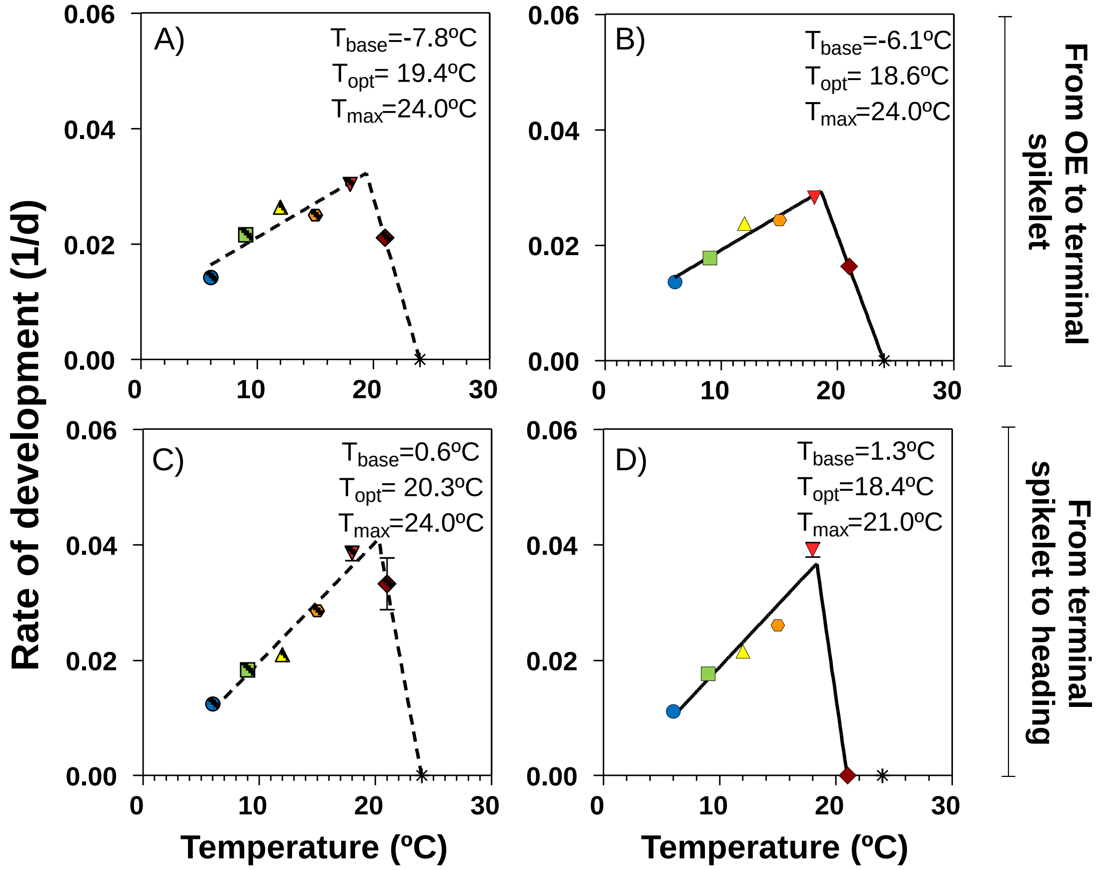

Supplementary Figure S3. Relationships between the rate of development between either from the onset of the experiment and terminal spikelet (A, B) or from then to heading (C, D) and temperature for lines carrying *Eps*-early (left panels) or *Eps*-late (right panels) alleles grown under constant temperatures of 6, 9, 12, 15, 18, 21 and 24°C. To avoid a biased estimate of parameters, lines were fitted by bi-linear regression fitted with data at temperatures between the base and the maximum thresholds (for lines carrying *Eps*-early 6-24°C and for *Eps*-late until 6-21°C). Insets are the calculated cardinal temperatures (base, T_{base}; optimum, T_{opt}; and maximum, T_{max}).
